# Proteomic characterization of novel histone post-translational modifications

**DOI:** 10.1186/1756-8935-6-24

**Published:** 2013-08-01

**Authors:** Anna M Arnaudo, Benjamin A Garcia

**Affiliations:** 1 Epigenetics Program, Department of Biochemistry and Biophysics, Perelman School of Medicine University of Pennsylvania, 1009C Stellar-Chance Laboratories, 422 Curie Boulevard, Philadelphia, PA 19104, USA; 2 Department of Molecular Biology, Princeton University, 119 Lewis Thomas Laboratory, Washington Road, Princeton, NJ 08544, USA

**Keywords:** Histone post-translational modifications, Mass spectrometry, Proteomics, Epigenetics

## Abstract

Histone post-translational modifications (PTMs) have been linked to a variety of biological processes and disease states, thus making their characterization a critical field of study. In the last 5 years, a number of novel sites and types of modifications have been discovered, greatly expanding the histone code. Mass spectrometric methods are essential for finding and validating histone PTMs. Additionally, novel proteomic, genomic and chemical biology tools have been developed to probe PTM function. In this snapshot review, proteomic tools for PTM identification and characterization will be discussed and an overview of PTMs found in the last 5 years will be provided.

## Review

### Introduction

Nearly 50 years ago Vincent Allfrey described histone acetylation [1]. Since then research has been focused on identifying and mapping a growing list of histone post-translational modifications (PTMs), including lysine acetylation, arginine and lysine methylation, phosphorylation, proline isomerization, ubiquitination (Ub), ADP ribosylation, arginine citrullination, SUMOylation, carbonylation and, with some controversy, biotinylation [2]. While PTMs are found on all five histones, they commonly map to histone N-terminal tails [3]. Functional characterization of these PTMs have implicated them in a variety of cellular processes including, but not limited to, transcription, DNA damage, apoptosis, and cell-cycle regulation [4]. In addition, histone-modifying enzymes are popular drug targets because they are misregulated in diseases such as cancer [5]. Histone PTMs impact biological processes in a number of ways. PTM acquisition or resulting changes in net charge can alter DNA-histone or inter-nucleosomal contacts, thereby modulating chromatin structure [6]. Alternatively, PTMs can act as a docking site for proteins containing specific structural domains - for example, chromodomains bind methylated lysines and bromodomains bind acetylated lysines [6,7]. The recruitment or repulsion of these proteins impacts downstream processes. The idea that PTMs constitute a code that is read by effector proteins is the basis for the histone code hypothesis [8,9]. Mass spectrometry (MS) has become an essential tool for deciphering this code, in part by identifying novel PTMs. In this review we will focus on MS and proteogenomic methods involved in identifying and characterizing novel sites and types of histone PTMs. Additionally, we will highlight the modifications that have been discovered in the last 5 years and have greatly added to the modifications listed above.

### Identification of novel post-translational modifications by mass spectrometry

In the search for novel modifications, MS has an advantage over other methods because no prior knowledge of the modification site or type is required. In traditional bottom-up analysis, proteins are digested to peptides with a protease like trypsin, peptides are separated using liquid chromatography (LC), subjected to MS for peptide identification, and then fragmented by MS/MS for peptide sequencing (for a basic review, see [10]). PTMs induce a mass shift (+14 Da for methyl, +42 Da for acetyl) that is detectable in the MS and MS/MS spectra [11]. Multiple software algorithms have been developed to detect and map modifications from MS and MS/MS data with varying degrees of success.

Analysis of histone modifications by conventional bottom-up MS techniques is challenging because histones are both lysine and arginine rich. Trypsin digestion results in short peptides that are incompatible with LC-MS and peptides of inconsistent length due to variable cleavage at modified residues (in other words, trypsin will cleave at mono-, but not di- or tri- methylated lysines) [12,13]. Chemical derivatization strategies can help overcome these challenges. Propionic anhydride derivatization results in cleavage only at the C-terminal of arginine, increasing sequence coverage and generating larger, consistently cleaved peptides [13]. These strategies therefore aid in discovering and quantifying histone PTMs. In addition to bottom-up strategies, middle-down and top-down strategies have been created to facilitate PTM discovery and explore combinatorial histone codes. Top-down utilizes whole histone protein [14], while middle-down utilizes alternative protease digestions to create large peptide fragments [15,16]. Both of these strategies rely on electron transfer dissociation (ETD), an MS/MS technique that is more suitable for fragmentation of highly charged, larger peptides [17].

Due to the highly modified nature of histones and the numerous PTM combinations that can result, reliably assigning modifications to histone peptides can be difficult. Algorithms have been designed to specifically map histone modifications and identify novel sites of modification [18-21]. For unbiased novel PTM type discovery, Chen and colleagues [18] developed PTMap to explore a wide window of mass shifts in small increments and identify unique modifications. It also decreases false positives by scoring unmatched peaks in the MS/MS spectra [18].

Misidentification of novel histone PTMs can come from a variety of sources including isobaric mass shifts due to histone sequence variation or other modifications, sample preparation, gas phase chemistry within the mass spectrometer, and false positives or incorrect database assignments [22]. In the case of methylation, for example, the use of methanol during sample processing can result in methylation of aspartic or glutamic acid [23]. Methyl transfer within a peptide has also been observed on singly charged peptides in the gas phase, which could ultimately result in a false positive methylation site assignment [24]. Modifications can also be lost during sample processing or MS analysis due to their chemical, enzymatic and/or MS/MS lability. Histidine phosphorylation was discovered on histones in the 1970s [25,26], but has been difficult to study by MS because it is acid labile and can be lost during sample processing [27]. Serine/threonine phosphorylation and O-glycosylation are susceptible to loss during sample preparation by enzymatic removal and during MS/MS fragmentation by collision induced dissociation [28,29]. Alternative fragmentation with ETD has been suggested to aid detection of labile modifications [17].

To be confident of a novel PTM, experimental confirmation is required. Heavy isotope labeling in cell culture with the modification donor can be used to confirm that it is acquired *in vivo* rather than during sample preparation. Such experiments have recently been used to probe sites of methylation using heavy methyl donor ^13^CD_3_- S-adenosylmethionine [22] and the presence of crotonylation using heavy D4 crotonate [30]. Pan-modification or site-specific antibodies are also commonly raised to test the *in vivo* presence of the modification [30]. Pan-modification antibodies have the added benefit that they can be used to probe all the core histones across multiple organisms. Traditionally, the standard for verification is the generation of a synthetic peptide containing the PTM of interest. MS/MS fragmentation and LC elution patterns from the *in vivo* derived peptide, the synthetic peptide and a mixture of the two are compared to confirm the site and type of modification [22,31]. Recent reports on the misidentification of serine methylation on histone H3S28 [22] and threonine acetylation on histone H2AT15 [31], however, indicate that synthetic peptides can be insufficient and suggest that further fragmentation (MS^3^) is necessary for confirmation.

### Novel sites and types of modifications

**Figure 1:**
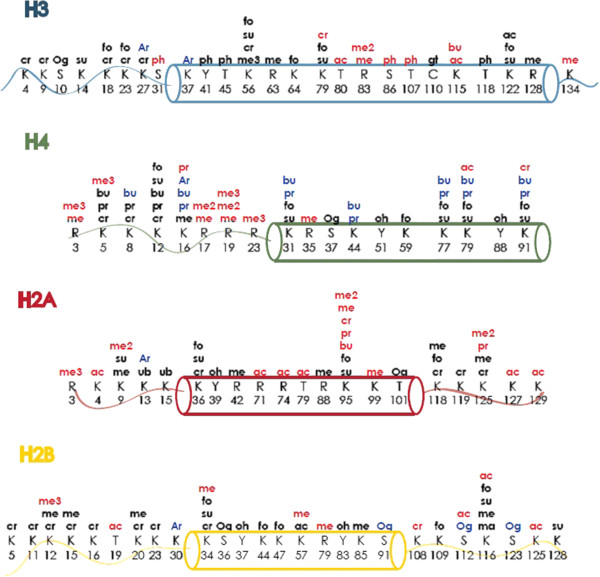
**Recently identified modifications on the core histones.** Black, modifications found *in vivo* in human; red, modifications found in mouse brain; blue, modifications found *in vitro*. ac, acetylation; Ar, ADP-ribosylation; bu, butyrylation; cr, crotonylation; fo, formylation; gt, glutathionylation; ma, malonylation; me, methylation; Og, O-glcNAcylation; oh, hydroxylation; pr, propionylation; su, succinylation; ph, phosphonylation; ub, ubiquitination.

In the last 5 years, a number of novel sites and types of PTMs have been discovered. A comprehensive list can be found in Figure 1. The novel types of modifications include tyrosine hydroxylation [30], serine and threonine acetylation [32], lysine crotonylation (Kcr) [30], lysine N-formylation [33], lysine succinylation [34], lysine malonylation [34], lysine propionylation [35], lysine butyrylation [35], O-GlcNAcylation (beta-N-acetylglucosamine) [36-38], lysine 5-hydroxylation [39] and cysteine glutathionylation [40]. Novel sites include Ub [41], phosphorylation [42,43], ADP-ribosylation [44], lysine acetylation and mono-, di- and tri- lysine methylations [30,45]. The degree of characterization varies for each PTM identified; however, there are some interesting findings and themes that emerge.

**Figure 2:**
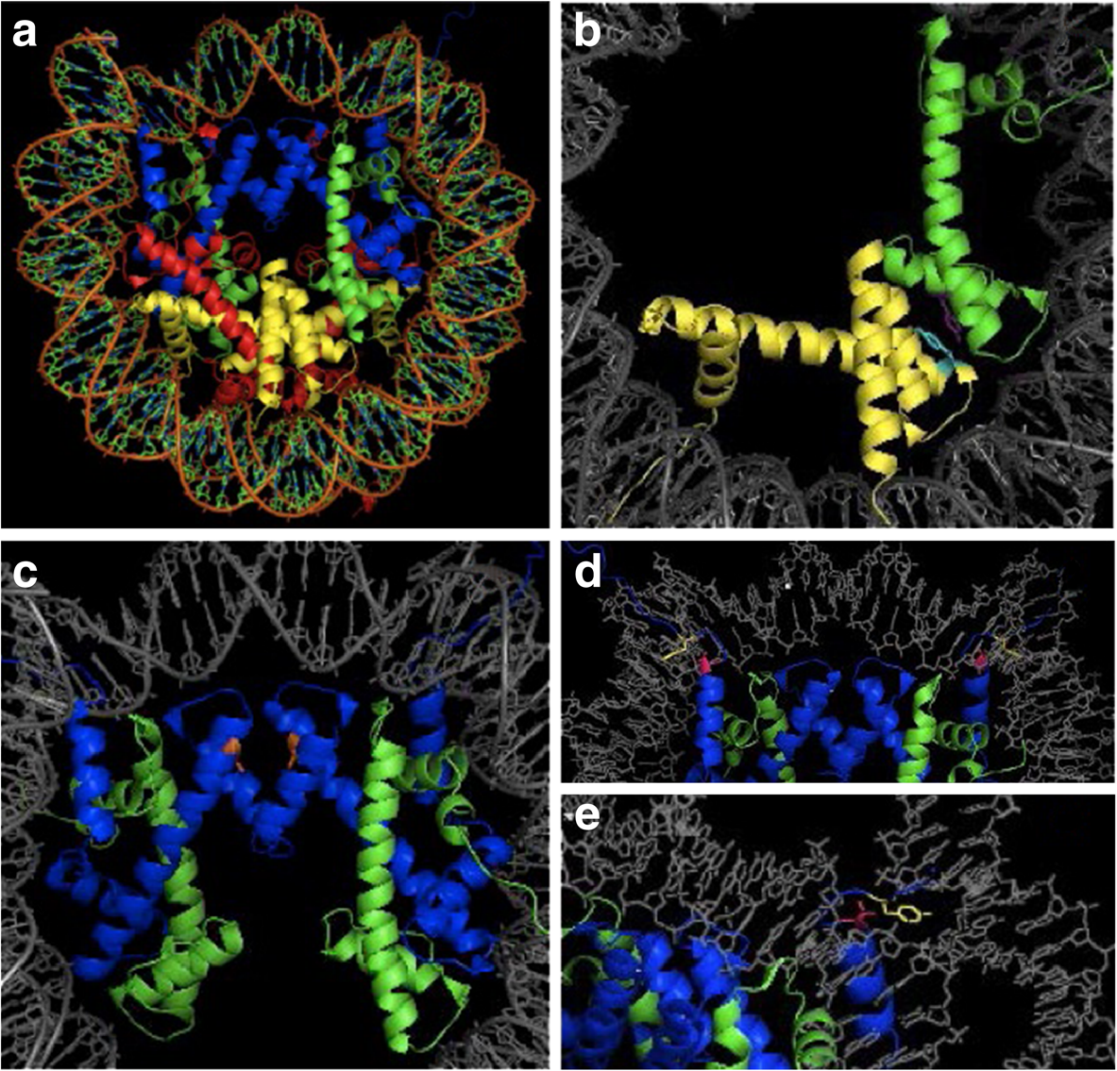
**Global domain post**-**translational modifications. (a)** The nucleosome with H3 (blue), H4 (green), H2A (red) and H2B (yellow). **(b)** Tryosine hydroxylation on H2BY83 (cyan) and H4Y88 (purple) occur at the H2B:H4 interface. **(c)** Glutathionylation of H3C110 mapped to the H3:H4 tetramer interface. **(d)** Phosphorylation on H3T45 (pink) and H3Y41 (yellow) mapped to the H3:H4 tetramer. **(e)** Phosphorylations from **(d)** occur near the H3:DNA contact.

One interesting trend is the prevalence of PTMs discovered in new areas of the histone protein. While canonical histone Ub is present in the C-termini of H2A and H2B, novel Ub sites have been mapped to the N-terminus of H2A at H2AK13 and K15. LC-MS/MS analysis confirmed the presence of Ub at both sites, which are contained on a single peptide. Functional analyses indicate these sites are present during the DNA damage response to double strand breaks and their modification is controlled by E3 ubiquitin ligase RNF168 [41]. A multitude of PTMs have been found on the histone globular domains, raising questions about how these modifications impact nucleosomal structure and stability Figure 2[3]. Tyrosine hydroxylation was identified on H2BY83 and H4Y88 in a MS screen for novel modifications using PTMap. Since these residues are located near the H2B-H4 contact, they may play a role in altering chromatin structure via intranucleosomal surface contacts [30]. Phosphorylation within the globular domain may also impact structure [43,46]. A novel site was identified at H3T45 by MS. Protein kinase C-gamma phosphorylation of this residue increases during apoptosis and any resulting change to nucleosomal structure may promote DNA fragmentation common to apoptosis [43]. Novel PTM glutathionylation of histone H3C110 was shown to destabilize nucleosomes by thermal stability tests [40]. Interestingly, global PTMs can also affect binding of reader proteins, a role typically associated with PTMs on histone tails. A novel phosphorylation site at H3Y41 was found with an antibody. Janus kinase 2 (JAK2) phosphorylates H3Y41, which when phosphorylated could act to ameliorate transcriptional repression of JAK2-controlled genes by preventing binding of HP1alpha [42,47].

A link between cellular metabolism and histone modification is another trend found within novel PTMs. N-formylation of lysine is thought to arise from oxidation. Deoxyribose oxidation can create a 3’-formylphosphate, which is free to attack lysine and create N-formyl-lysine [33]. LC-MS/MS analysis using high mass accuracy to differentiate formylation and di-methylation shows that N-formyl-lysine can occur on all five histones, at sites that are commonly methylated or acetylated [48]. Competition of N-formylation with other modifications for lysine residues could be a way DNA oxidation resulting from cellular metabolism can impact PTM-mediated cellular processes [33]. Another way that metabolism affects PTM acquisition is through the use of different coenzyme A (CoA) molecules as PTM donors. This link has been well established for acetyl-CoA and lysine acetylation. More recently, lysine propionylation and butyrylation were identified on histone H4 by LC-MS/MS and confirmed using synthetic peptides. *In vitro* experiments showed that known histone acetyltransferases (HATs) [46] CBP and p300 lysine were able to catalyze reactions using radioactive propionyl-CoA and butyryl-CoA as donors for lysine propionylation and butyrylation, respectively. Since propionyl-CoA and butyryl-CoA are derived from different metabolic processes and levels fluctuate across different physiological conditions, Chen and colleagues suggest that these modifications may play a role in regulating cellular metabolism [35]. A separate study also identified lysine succinylation and lysine malonylation, using affinity enrichment with anti-succinyllysine and anti-malonyllysine antibodies in combination with LC-MS/MS analysis. Since succinyl-CoA and malonyl-CoA are also metabolic intermediates, these may also link metabolism with histone PTMs [34]. Further studies on all these CoA-related PTMs are needed to determine how they are established *in vivo* and what processes they regulate.

One novel lysine modification, Kcr, has been relatively well characterized. It was discovered by a characteristic 68 Da mass shift by LC-MS analysis and confirmed using synthetic peptides. In the genome, Kcr localizes to potential enhancers and to promoters, showing enrichment at transcription start sites [30]. One functional role for this modification is in haploid male germ cell differentiation. Kcr was found at active genes on the sex chromosomes in haploid spermatids, and its presence was dependent upon histone H2A ubiquitin ligase RNF8. The fact that transcription of sex chromosomes is downregulated at this stage indicates that Kcr may be part of an epigenetic program that protects a subset of genes from repression [30,49,50]. Western blot analysis of histones from mouse, *Drosophila*, *Caenorhabditis Elegans*, and *Saccharomyces cerevisiae* indicate that this modification is conserved, raising the question as to what role this modification may play in these organisms [30]. O-GlcNAcylation of histones has also been studied heavily [36-38]. Sites have been identified on serines or threonines on all four core histones using MS, antibodies or lectin. It may be involved in a couple of cellular processes, given that levels increase during recovery from heat shock and levels fluctuate in a cell-cyle-dependent manner [37]. The finding of GlyNAcylation on H3S10, a residue commonly associated with cell-cycle, may be particularly interesting [38]. O-GlcNAcylation also may be important for crosstalk with other modifications; H2BS112GlyNAc was found to promote H2B120Ub in *Drosophila*[36].

The possibility of numerous modifications occurring on a single residue, like the preponderance of lysine PTMs discussed above, raises questions about how these modifications are modulated either temporally or physically to create a functional readout. Lysine residues susceptible to ADP-ribosylation were identified with *in vitro* ribosylation reactions and subsequent ETD fragmentation of histone peptides. Residues in the N-termini of all four core histones were found to be ADP-ribosylated, including histone H4K16. Acetylation of H4K16 impeded *in vitro* ribosylation at this residue, indicating a competitive relationship between these modifications [44]. Newly identified lysine 5-hydroxylation can block acetylation and methylation by HAT p300 and methyltransferase SMYD3 during *in vitro* reactions [39]. A similar interplay may occur at serine/threonine residues, where phosphorylation and O-GlcNAcylation reside. Serine/threonine acetylation was discovered in adult mouse brain by MS analysis and, although little is known, it may be of interest due to its ability to compete with the other modifications at these residues [32].

### Characterization of novel post-translational modifications

As seen above, the discovery of a new modification raises a variety of questions, including: (1) is the modification evolutionarily conserved, (2) where in the genome is it localized [51], how abundant is it, (4) does it occur in the tails or globular domain of the histone, (5) what enzymes are responsible for acquisition/removal, (6) does it cross-talk with previously known modifications or histone variants, (7) does it antagonize other PTMs or abrogate binding of their reader proteins, and (8) what proteins bind or 'read' the modification? The answers to these questions lend insight into the biological function of the PTM. Evolutionary conservation across species, for example, indicates that it could be essential for a conserved cellular process. Abundance of a modification, on the other hand, may not be as indicative of its importance since it has been observed that low-level modifications like H3K4me3 play vital roles in biological processes such as transcription [52]. The neighboring chromatin environment and the genomic localization of modifications to distinct regions may give more valuable information toward gauging relevance and function.

New proteomic, genomic and chemical biology technologies have been developed or proposed to address the above questions [7,12,53]. Analysis of DNA from chromatin immunoprecipitation (ChIP) by high-throughput sequencing has become a standard tool for assessing PTM localization within the genome [54]. More recently, native ChIP methodologies have been developed to allow for isolation and quantitative PTM analysis of histone proteins, a technique referred to as chromatin immunoprecipitation with quantitative MS (ChIP-qMS) [51,55,56]. Native ChIPs can be performed with either a reader protein or with a PTM-specific antibody to obtain the associated histone codes and histone variants. For example, FLAG-tagged bromodomain-containing Brd proteins and chromodomain-containing HP1 proteins were immunoprecipitated and the associated histone was analyzed by MS. As expected, histones from Brd ChIPs were enriched for active marks, while histones from HP1 chips were enriched for silencing marks [56]. PTM ChIPs recently demonstrated that nucleosomes can be asymmetrically modified, meaning that only one tail within the octamer is modified. In the case of H3K36me3/H3K4me3, symmetrical modification of both tails seems to prevent PRC2 activity on H3K27, while asymmetric modification allows for PRC2 activity resulting in H3K27me3 on the opposing tail. The existence of asymmetrical modifications adds another layer of complexity to the histone code [51]. ChIP-qMS technologies have not been utilized for novel PTMs to date and, due to their reliance on antibodies or tagged constructs, these experiments are limited to known chromatin-associated proteins or PTMs. While they can be viewed as an improvement to whole genome quantitative PTM experiments, they are still unable to focus on a particular chromatin locus or region.

In an attempt to surmount these limitations, Dejardin and Kingston [57] and Byrum and colleagues [58] implemented distinct methods for isolating specific genomic loci and used MS to identify loci-specific proteins and modified histone forms. Byrum and colleagues’ chromatin affinity purification with MS method used a Lex-A binding site in the *GAL1* locus of yeast to facilitate purification of this locus and its associated proteins/histones during silent and active states [58]. Dejardin and Kingston’s proteomics of isolated chromatin method hybridized a desthiobiotin labeled oligonucleotide to telomeric DNA allowing for MS identification of telomere-associated proteins in mammalian cells [57]. Capture of known telomere-associated proteins using this protocol indicates this is a valid approach for identifying chromatin-associated proteins. These methods that can purify small regions of chromatin may be helpful in the discovery of novel low-level PTMs or PTMs that are restricted to particular areas of the genome, both of which may be hard to detect in whole genome analyses. They may also aid in identifying new reader proteins.

To identify reader proteins that bind to specific modifications, synthetically modified nucleosomes generated by native protein ligation have been used as bait for reader proteins. These stable isotope labeling of amino acids in cell culture (SILAC) nucleosome affinity purification experiments utilize SILAC labeling to compare proteins bound to synthetic nucleosomes. Unmodified and modified nucleosomes are incubated in light and heavy SILAC-labeled nuclear lysates, respectively, and the proteins isolated are mixed one to one. The light/heavy ratio tells which proteins preferentially bind the modified nucleosome and may act as a reader. Interestingly, these data also provide insights into what proteins PTMs may prevent from binding [59].

Most recently, SILAC labeling has also been utilized in yeast to examine modifications that potentially crosstalk with modifications on either H3K79 or H3K56. Yeast mutant strains with amino acid substitutions to mimic modified states were grown alongside wild-type yeast, one of which was grown in heavy media. By comparing heavy/light ratios from a mutant and wild-type pair, the impact of the modified state on H3K79 and H3K56 modifications could be determined. The analyses indicate that modifications on all four core histones can effect positively or negatively the modification levels on H3K56 and H3K79 [60].

## Conclusions

A review of the recent literature reveals that novel sites or types of histone PTMs are rapidly being discovered and characterized, in part due to the powers of MS analysis and emerging proteomic, genomic and chemical biology tools. The diversity seen in terms of location on the nucleosome, genome localization and the cellular processes in which they are involved highlight the importance of histone PTMs to multiple fields of study including cell biology, epigenetics, development and cancer biology. Since many of these modifications remain poorly characterized, their discoveries open up new avenues of research and promote the development of novel technologies. The sheer number of novel modifications begs the question how many more types of PTMs are there remaining to be found?

## Abbreviations

ChIP: Chromatin immunoprecipitation; ChIP-qMS: Chromatin immunoprecipitation with quantitative mass spectrometry; Co-A: Coenzyme A; ETD: Electron transfer dissociation; HAT: Histone acetyltransferase; Jak2: Janus kinase 2; Kcr: Kysine crotonylation; LC: Liquid chromatography; MS: Mass spectrometry; PTM: Post-translational modification; SILAC: Stable isotope labeling of amino acids in cell culture; Ub: Ubiquitination.

## Competing interests

The authors declare that they have no competing interests.

## Authors’ contribution

AMA contributed to content, wrote the manuscript and created the figures. BAG revised and contributed to content of the manuscript. Both authors read and approved the final manuscript.
